# Usefulness of Video Head Impulse Test Results in the Identification of Meniere's Disease

**DOI:** 10.3389/fneur.2020.581527

**Published:** 2020-10-29

**Authors:** Brahim Kaci, Mujda Nooristani, Tamara Mijovic, Maxime Maheu

**Affiliations:** ^1^Vestibulab, School of Speech Language Pathology and Audiology, University of Montreal, Montreal, QC, Canada; ^2^Centre de Recherche Interdisciplinaire en Réadaptation - Institut Universitaire sur la Réadaptation en Déficience Physique de Montréal (IURDPM), Pavillon Laurier, CIUSSS du Centre-Sud-de-l'Île-de-Montréal, Montreal, QC, Canada; ^3^Department of Otolaryngology-Head and Neck Surgery, Royal Victoria Hospital, Montreal, QC, Canada

**Keywords:** Meniere disease, vestibular system, endolymphatic hydrops, caloric, vHIT

## Abstract

Meniere's disease (MD) is an inner ear disorder inducing tinnitus, aural fullness, sensorineural hearing loss, and vertigo episodes. In the past few years, efforts have been made to develop objective measures able to distinguish MD from other pathologies. Indeed, some authors investigated electrophysiological measures, such as electrocochleography and vestibular evoked myogenic potentials or imaging techniques. More recently, the video head impulse test (vHIT) was developed to assess the vestibulo-ocular reflex (VOR). In the last few years, authors aimed at identifying how vHIT may help to identify MD. The objective of this manuscript is to review the different vHIT results in MD patients. We will discuss the usefulness of these findings in the identification of MD, how these results may be explained by pathophysiological mechanisms associated with MD, and finally provide directions for future studies.

## Introduction

Meniere's disease (MD) is an inner ear pathology that induces episodes of vertigo, ear fullness, tinnitus, and fluctuating sensorineural hearing loss. A commonly accepted pathophysiological mechanism of MD is endolymphatic hydrops (EH) (1). Indeed, EH is a disorder where the endolymph accumulates in the cochlea and in the vestibular organs (2) and is revealed to be a frequent finding in histopathological and imaging studies of the inner ear of MD patients (3). The American Academy of Otolaryngology—Head and Neck Surgery (AAO-HNS) (4) has defined the diagnostic criteria that are based on the presence/absence and duration of the different symptoms described above allowing the classification of patients in different MD categories, such as probable, possible, definite, and certain. These criteria and the classification have been reviewed in 2015 with the idea to improve diagnosis, leaving only probable and definite MD (5). However, the diagnosis of MD remains difficult probably due to the lack of a gold standard test.

In the past few years efforts have been made to develop an objective test able to accurately identify MD. Previous studies aimed at identifying MD markers either at the cochlear level or at the vestibular level using, respectively, electrocochleography and vestibular evoked myogenic potentials. No consensus could be reached from these studies regarding their efficacy in the identification of MD [for review, see (6–8)]. More recently, the video head impulse test (vHIT) was developed to assess the vestibulo-ocular reflex (VOR), and efforts have been made to understand how MD influences vHIT results. However, studies are sometimes contradictory as some found reduced VOR results, whereas others found enhanced VOR results. Moreover, contradictory findings have been observed between vHIT and caloric test, both assessing horizontal canal function. The objective of this manuscript is to review the different vHIT results in MD patients. We will discuss the usefulness of these findings in the identification of MD, how these seemingly contradictory results may be unified, and how they relate to the pathophysiological mechanisms associated with MD. Finally, we will provide directions for future studies.

## Video Head Impulse Results in Meniere

### Video Head Impulse Test

The vHIT is a recently developed tool allowing the assessment of high-frequency VOR function of all six semicircular canals (9). It uses a gyroscope and an infrared camera mounted on goggles to record, respectively, head and eye velocity. With these parameters, it is possible to calculate VOR gain (eye/head) and to detect catch-up saccades during fast and passively applied head impulses (10). During these impulses, participants are required to maintain gaze on an earth-fixed target. It is assumed that when VOR is normal, the eyes will move at the same velocity as the head but in the opposite direction generating a perfect VOR gain of 1.0. However, when VOR is abnormal, the eyes will follow the head's movement reducing the VOR gain and requiring catch-up saccades to bring gaze back on target (10).

### Reduced VOR Function in MD

Some of the previous studies investigated the distribution of the abnormal results across all six semicircular canals in the quiescent phase (interictal) and found divergent results (Table 1). In 2014, Zulueta-Santos et al. (13) studied 36 participants with definite MD and revealed that the most frequently affected canal in MD participants was the posterior (40%), followed by the superior (22.8%) and the lateral (14.2%). More recently, Sobhy et al. (14) studied 40 patients with definite MD and observed the exact opposite classification. This group observed that the most frequently affected canal was the lateral (20%), followed by the superior (7.5%) and the posterior (5%). The possible differences observed between these studies could be, at least in part, due to two methodological differences in the definition of abnormal values. First, Zulueta-Santos et al. (13) used only the VOR gain to classify results as abnormal (normal if VOR gain was: <0.8 for lateral canals and <0.7 for vertical canals). On the other hand, Sobhy et al. (14) as well used VOR gain (absolute and asymmetry) but also included the presence of catch-up saccades to classify results as normal or abnormal. The latter method is in line with the results reported by Jerin et al. (17). This group studied 54 participants with certain MD and found that the vast majority (74%) of the participants showed normal VOR gain but presented catch-up saccades. These saccades were present in <50% of the trials in 14 out of 54 participants and in at least 50% of the trials in 33 out of 54 participants. Therefore, it seems that catch-up saccades are an important component to take into consideration and it could influence the categorization of a normal or abnormal VOR function. Second, another methodological discrepancy between these two studies could be the normative gain value applied. Indeed, contrary to Zulueta-Santos et al. (13), Sobhy et al. (14) did not specify their cut-off value for normal and abnormal VOR gain limiting possible comparisons.

**Table 1:** Comparison of the reviewed reports that investigated the influence of MD on vHIT results (gain and saccades).

**MD diagnosis and duration**	***N***	**Normative values (vHIT)**	**Results**	**Moment of evaluation**	**References**
Diagnosis: definite MD^a^ Duration: 0.5–4 years	24	Lateral: 0.88–1.27 Anterior: 0.75–1.29 Posterior: 0.77–1.13	Irritative/recovery phase: reduced gain in 37% Paretic phase: reduced gain in 79%	Ictal phase (irritative/recovery and paretic phases)	(11)
Diagnosis: N/A Duration: 2 years	1	N/A	Paretic phase: reduced gain Interictal: gain returned to baseline	Ictal phase (paretic phase) Outside the ictal phase	(12)
Diagnosis: definite MD^b^ Duration: 4–9 years	36	Lateral: >0.8 Anterior: >0.7 Posterior: >0.7	Lateral: 14.2% Anterior: 22.8% Posterior: 40%	Outside the ictal phase	(13)
Diagnosis: definite MD^b^ Duration: 3 months−15 years	40	Lateral: 1.03 ± 0.12 Anterior: 0.95 ± 0.11 Posterior: 1.03 ± 0.18	Lateral: normal 80%/enhanced 7.5%/reduced 12.5% Anterior: normal 95%/reduced 7.5% Posterior: normal 92.5%/reduced 5%	Outside the ictal phase	(14)
*Case 1* Diagnosis: probable MD^a^ Duration: 5 years *Case 2* Diagnosis: definite MD^a^ Duration: 10 years	2	N/A	Case 1: enhanced VOR gain Case 2: enhanced VOR gain	Case 1: N/A Case 2: 10 days after the last episode	(15)
Diagnosis: definite MD^a^ Duration: 1–11 years	10	Lateral: >0.8 or <1.11	Interictal: no difference between affected and unaffected, controls Ictal: reduced gain between affected and unaffected/controls	Outside the ictal phase Ictal phase (initial, peak, recovery) Post-attack	(16)

a *Bárány Society criteria (2015)*.

b *American Academy of Otolaryngology—Head and Neck surgery criteria (1995) (AAO-HNS)*.

### Fluctuation of VOR Function in MD

As MD being an episodic disorder, some authors measured VOR parameters at different moments of the MD episodes (before, during, or in between episodes) (Table 1). The vHIT findings seemed to fluctuate depending on the moment of the evaluation. Yacovino and Finlay (12) followed up a 64-year-old patient with MD over a 12-month period. They measured vHIT during and between episodes of vertigo. They noticed that during the episodes, VOR gain was reduced, and saccades were present on the affected side. When tested between episodes, patients showed normal gain and either fewer or no catch-up saccades. The contralateral side remained stable over time. This fluctuation in VOR gain was also observed in a 54-year-old patient known for MD. They measured VOR gain and saccades using vHIT before, during, and after an MD episode. They found similar results (reduced gain during an episode and a return to normal after a crisis) but only for the superior canal of the affected ear (18). Yacovino et al. (16) recently demonstrated this variation in the different phases of MD episodes (initial, peak, and recovery) in 10 patients. They showed a significant reduction in VOR gain between the affected and unaffected ears for all three different phases. However, no gain differences were observed between the affected and unaffected ears between episodes (interictal) and post-attack. Interestingly, these studies reveal different results as opposed to Rey-Martinez et al. (15), who report enhanced VOR gain in two patients known with MD. This group described the case of a 74-year-old male with probable MD and the case of a 45-year-old female with definite bilateral MD. The first case described was a male subject followed up for a period of 5-years, and they found systematically enhanced VOR gain. In this case, the authors did not report if the assessment was done during or between crisis. The second case was evaluated once, 10 days following the episode, and they also found an enhanced VOR gain.

## vHIT vs. Caloric

### Caloric Stimulation

VOR can be examined using different methods, such as vHIT and caloric test. vHIT is known to evaluate VOR at frequencies between 5 and 7 Hz (19, 20) and caloric between 0.003 and 0.008 Hz (21). Caloric test is thought to induce an endolymphatic movement in the horizontal semicircular canal. It consists of increasing or decreasing the temperature of the external auditory canal, using air or water, to induce a movement of the endolymph in the horizontal semicircular canal. This movement modulates the spontaneous firing rate of the peripheral vestibular system and creates an asymmetry in the plane of the horizontal semicircular canal (21).

### Discrepancies Between Caloric Test and vHIT in Meniere's Disease

Over the past few years, there has been a growing interest in observing how caloric test and vHIT are differently affected by MD as these two tests evaluate the function of the horizontal semicircular canals but tend to show opposing results in MD. As demonstrated in Table 2, it has been noticed by previous studies that even though caloric test usually demonstrates an asymmetry toward the affected ear, vHIT parameters are usually preserved in definite MD (22–30). Some authors tried to evaluate if this discrepancy in the results may be in relation to the duration or the stage of MD but failed to find a significant relationship (25, 27, 29). Limviriyakul et al. (29) went further and assessed if this discrepancy was related to patients being in the active or inactive phase according to the Shea classification of MD (31). These authors separated the participants into two groups, either active (symptoms within the past 3 months) or inactive (symptom free for at least 3 months). However, this study failed to reveal any significant difference between these two groups regarding the number of participants with and without caloric/vHIT discrepancies.

**Table 2:** Comparison of the reviewed reports that investigated the discrepancy of caloric and vHIT results in MD.

**MD diagnosis and duration**	***N***	**Normative values (caloric)**	**Normative values (vHIT)**	***N* (%) CV**	**Moment of evaluation**	**References**
Definite MD^a^ Mean duration: N/A	3	Abnormal: unilateral weakness (UW) >22% and directional preponderance (DP) >28%	Abnormal: VOR gain <0.7 and/or saccades	3 (100%)	No indication of patients' state (ictal or outside the ictal phase) during vestibular assessment nor MD stages	(22)
Definite MD^b^ Mean duration: 3 years	32	Abnormal: sum of slow phase velocity (SPV) of one ear (cold and warm) <5°/s (unilateral) or sum of SPV (cold and warm) of both ears <12°/s (bilateral)	Abnormal: presence of saccades and/or VOR gain <0.8 for lateral SCCs VOR gain <0.75 for vertical SCCs	17 (43.6%)	Outside the ictal phase: at least 72 h after MD crisis	(23)
Definite MD^b^ Mean duration: 7 years	37	Abnormal: UW ≥20%	Abnormal: presence of saccades or VOR <0.78 for lateral SCCs VOR <0.64 for vertical SCCs	Total: 34 (91.9%) Patients outside MD crisis: 31 (94%) Patients just after MD crisis: 3 (75%)	Outside the ictal phase: 33 patients Just after an MD crisis (irritative/recovery phase): 4 patients	(24)
Definite MD^a^ Mean duration: 5 ± 6.2 years	87	Abnormal: vestibular preponderance >22% or DP >28% or Irrigation response V_max_ <15°/s	Abnormal: VOR gain <0.8 and saccades	132 ears (86.8%)	Outside the ictal phase	(25)
Definite MD^a^ Mean duration: 3 ± 2.3 years	88	Abnormal: UW >20% and DP >28%	Abnormal: VOR gain <0.8 for lateral SCCs VOR gain <0.7 for vertical SCCs and saccades	Lateral canal: 40 (45.6%) All 6 canals: 16 (18.2%)	Outside the ictal phase	(26)
Definite MD^b^ Mean duration: N/A	73	Abnormal: UW ≥30% or Bilateral slow phase velocity responses were ≤ 10°/s	Abnormal if VOR <0.8	27 (37.0%)	Outside the ictal phase	(27)
MD classification not indicated^b^ Mean duration: N/A	20 (15 unilateral, 5 bilateral; total: 25 ears)	Abnormal: ENG response ≤ 10°/s.	Abnormal: VOR gain <0.8 for lateral SCCs or VOR gain <0.8 and saccades	14/25 ears (56%) 14/20 MD patients (70%)	No indication of patients' state (during or after crisis) during vestibular assessment	(28)
Definite MD^a^ Mean duration: N/A	51	Abnormal: UW >25%	Abnormal: VOR gain <0.8 for lateral SCCs VOR <0.7 for vertical SCCs or Presence of overt and covert saccades for >50% of trials	Active: 16 (40%) Inactive: 6 (54.6%)	Shea classification of MD: Active (symptoms within the past 3 months): 40 Inactive (symptom free for at least 3 months): 11	(29)

a *American Academy of Otolaryngology—Head and Neck surgery criteria (1995) (AAO-HNS)*.

b *Bárány Society criteria (2015)*.

There seems to have a higher prevalence of caloric/vHIT discrepancy in MD patients as opposed to controls when the diagnosis is based on the criteria proposed by AAO-HNS (1995) (4) or on the Classification Committee of the Bárány Society (2015) (5). This leads to propose that the presence of an abnormal caloric response in the presence of a normal vHIT results may be used as a marker in the diagnosis of MD. However, a recent study raised doubts as they failed to find an association between caloric/vHIT discrepancy and the pathophysiological marker of MD, namely, EH (28). They used an inner ear MRI to classify MD patients based on the presence or absence of a positive sign of EH. They sought to compare the number of patients with opposing caloric and vHIT results in each group and found that a positive finding of EH demonstrated by inner ear MRI did not always indicate a caloric/vHIT discrepancy in an MD group. More precisely, they observed that out of 29 patients who showed a discrepancy in caloric and vHIT results, 15 showed a positive sign of EH as opposed to 14 who had a negative sign of EH. Instead, they found that the discrepancy between caloric and vHIT results was strongly associated with vestibular herniation into the semicircular canals (protrusion of part of the vestibular labyrinth into the semicircular canals).

## Discussion

This review aimed at presenting the different vHIT findings observed in MD. The results of the reviewed studies may seem to be heterogeneous as some demonstrated that VOR is well-preserved, whereas others reported a reduced VOR gain with saccades or even an enhanced VOR gain. However, we propose that these results may be unified, at least in part, when considering the moment of assessment relative to the ictal phase or the duration of the disease.

First, MD, being an episodic disease, comprises two phases, either ictal (during MD episode) or outside the ictal. It is documented that the ictal phase could be subcategorized into three phases: (1) irritative, (2) paretic, and (3) recovery. The irritative and recovery phases are characterized by spontaneous nystagmus that beats toward the affected ear as opposed to the paretic phase that is characterized by a nystagmus beating toward the unaffected ear (32). Based on the studies reported in this review, when patients are tested during the ictal paretic phase, the VOR gain associated with the affected ear is usually reduced (11, 12). However, when patients are assessed during either the irritative or recovery phase of the ictal period or assessed outside the ictal period, the majority of the MD patients demonstrate a normal VOR gain. These results associated to the different phases of MD are supported by animal model of EH. Indeed, EH has been associated with increase in perilymph potassium concentration (33). In animal models, induction of potassium within the tympanic cavity revealed similar irritative and paretic phases seen in MD patients (34). Therefore, these pathophysiologic evidences support that the fluctuations in VOR gain measured by vHIT could be an indicator of EH.

Second, even though no association between duration of the disease and VOR gain was revealed in the reviewed studies (25), previous literature suggests an interaction between duration of the disease and moment of the evaluation (ictal phase or outside the ictal phase). Indeed, Maire and Van Melle (35) observed a dissociation of VOR response between early (<12 months) and late (>12 months) MD participants using a rotary chair. They found that early and late MD participants showed opposite results during the ictal phase. While early MD revealed enhanced VOR, late MD showed reduced VOR gain. This may explain differences with van Esch et al.'s (25) study that assessed participants between MD episodes (outside the ictal phase). To our knowledge, no studies using vHIT assessed the relationship between MD phase and MD duration. Therefore, it would be of great interest for future studies to investigate vHIT results in early and late MD for all six semicircular canals and separate groups in relation to the MD phase (during or between MD episodes). This could help to advance our understanding of the pathophysiology of MD and its influence on vHIT results.

The present manuscript also reviewed research that studied the caloric/vHIT discrepancy, which seems to be a promising indicator of MD. However, some questions remain unanswered. One question that future studies would need to assess is if this discrepancy occurs only at a certain moment in the progression of MD. Indeed, as described by Sugimoto et al. (36), vestibular herniation is thought to explain the discrepancy between caloric and vHIT results and is associated with the progression of EH. This could lead to hypothesize that the discrepancy between caloric test and vHIT may more frequently occur only at a certain stage/duration of the disease. To our knowledge, no previous studies investigated this question, and therefore, future studies should examine this hypothesis by analyzing the ratio of caloric and vHIT discrepancy in relation to the different stages and durations of MD. Furthermore, the differences between the caloric/vHIT discrepancies may be related to the phase of MD. When analyzed closely, the percentage of patients showing caloric/vHIT discrepancy is higher when they were assessed outside the ictal phase than when they were assessed during the ictal phase (Table 2). This could be related to the previous observation that during the ictal paretic phase, VOR gain is decreased that may reduce the percentage of MD patients showing caloric/vHIT discrepancy.

In the past few years, several theories have been put forward to explain how the pathophysiology of MD may explain the caloric/vHIT discrepancy. One of the theories, well-described in McCaslin et al. (22), states that MD differently affects regular and irregular afferents. Indeed, a significant loss of type II hair cells in the crista with MD has been reported, suggesting that MD specifically damages regular afferents. This theory is based on the fact that peripheral zones of the crista are characterized by afferents that are most sensitive to low-frequency movements (regular afferents) as opposed to afferents located centrally that are most sensitive to higher frequencies (irregular afferents). Therefore, as described earlier, the caloric test (low frequency) will stimulate the regular afferent of the semicircular canal that responds to sustained stimulation as opposed to the transient high acceleration head impulses delivered during vHIT examination, which will mostly stimulate the irregular afferents (37, 38). However, some doubts are raised about the fact that MD affects specifically regular afferents as a study found that both type I and type II hair cells were equally affected in MD (39). A second theory was put forward by Mcgarvie et al. (40). This theory is based on the physiology of the canal and the impact of EH. Mcgarvie et al. (40) suggest that the caloric weakness observed in MD is due to the hydropic expansion of the lateral canal membranous duct. The enlargement of the canal would reduce the hydrostatic drive, normally provided by a change in endolymph temperature, by inducing local convection flows and thus reducing slow phase eye velocity when the affected canal is being stimulated with the caloric test. However, the hydrops would not reduce the endolymphatic flow generated following head acceleration and, therefore, would not affect VOR gain. This theory recently received support from Fukushima et al.'s (41) study that observed that endolymphatic volume affects caloric response but is independent of the vHIT results. Therefore, this theory states that the discrepancy between the results may be due to the physical enlargement of the labyrinth due to EH.

Some important points may limit the comparisons of the studies reviewed here and need to be highlighted. First, the lack of description of the inclusion/exclusion criteria related to medication/treatment. This is of great importance as the spectrum of possible treatment for MD varies a lot and may have different impacts on vestibular measures [for a review of possible treatments, see (42)]. Future studies should always report the medication/treatment received by the participants. Second, an important variation in the normative criteria used to identify a vestibular dysfunction between studies was noted. More specifically, the normative caloric asymmetry ratio used by the different studies reviewed varied between 20 and 30%. This variation seems to be associated with the variation of caloric/vHIT discrepancies. Generally, greater cut-off values are associated with lower discrepancy ratios, and inversely, higher discrepancy ratios are observed with lower cut-off values. Further research should aim at identifying the optimal cut-off values for caloric test and vHIT to improve the accuracy of the discrepancy between these two tests before suggesting this approach as a diagnostic indicator of MD.

Finally, recent evidence reported in this review cannot support the sole use of vHIT in the identification of MD. However, it would be of great interest for future studies to assess the high-frequency VOR fluctuation in seriated measures as opposed to only cross-sectional. Future studies will also need to determine the optimal parameters (gain vs. saccades) and moments of evaluation (ictal vs. outside the ictal). However, for now, vHIT is a valuable complement in the evaluation of MD along with the caloric test as the dissociation between these two tests is a promising indicator in the identification of MD.

## Author Contributions

BK, MN, and MM reviewed the literature. BK, MN, TM, and MM wrote the paper. All authors discussed the results and implications and commented on the manuscript at all stages.

## Conflict of Interest

The authors declare that the research was conducted in the absence of any commercial or financial relationships that could be construed as a potential conflict of interest.
